# Microstructure of Coatings on Nickel and Steel Platelets Obtained by Co-Milling with NiAl and CrB_2_ Powders

**DOI:** 10.3390/ma12162593

**Published:** 2019-08-15

**Authors:** Maciej Szlezynger, Jerzy Morgiel, Łukasz Maj, Olena Poliarus, Paweł Czaja

**Affiliations:** 1 Institute of Metallurgy and Materials Science, Polish Academy of Sciences, 25 Reymonta st., 30-059 Kraków, Poland; 2 Frantsevich Institute for Problems of Materials Science, National Academy of Sciences of Ukraine (IPMS NASU), 3 Krzhyzhanovsky st., 03142 Kyiv, Ukraine

**Keywords:** NiAl, mechanical alloying, composite coatings, metal-matrix composites

## Abstract

Metal matrix composite coatings are developed to protect parts made from materials susceptible to wear, like nickel alloys or stainless steel. The industry-established deposition method is presently an atmospheric plasma spraying method since it allows the production of both well-adhering and thick coatings. Alternatively, similar coatings could be produced by co-milling of ceramic and alloyed powders together with metallic plates serving as substrates. It results in mechanical embedding of the powder particles into exposed metallic surfaces required coatings. The present experiment was aimed at the analysis of microstructure of such coatings obtained using NiAl and CrB_2_ powders. They were loaded together with nickel and stainless steel platelets into ball mill vials and rotated at 350 rpm for up to 32 h. This helped to produce coatings of a thickness up to ~40 µm. The optical, scanning, and transmission electron microscopy observations of the coatings led to conclusion that the higher the rotation speed of vials, the wider the intermixing zone between the coating and the substrate. Simultaneously, it was established that the total thickness of the coating deposited at specified conditions is limited by the brittleness of its nanocrystalline matrix. An increase in the hardness of the substrate results in a decrease of the intermixing zone. The above results indicate that even as the method based on mechanical embedding could so far produce thinner coatings than the plasma spraying, in the former case they are characterized by a more uniform nanocrystalline matrix with homogenously distributed fine ceramic particles.

## 1. Introduction

Metal-matrix composite (MMC) coatings might allow improving the surface properties of materials, but eventual success of such operations depends on a proper choice of matrix and strengthening phase, as well as a careful design of their microstructure. The NiAl matrix offers a number of advantages, including high hardness and oxidations resistance, allowing to protect parts used in the aviation industry, especially in gas turbine engines [1,2,3]. However, at elevated temperatures it softens and makes itself more and more susceptible to abrasion. In order to eliminate the latter drawback, numerous borides including TiB_2_, ZrB_2_, or CrB_2_ with up to 30 vol% were tried as strengthening phases for NiAl matrix [4,5]. Eventually, the best improvement in wear resistance at 500 °C showed coatings with 15% CrB_2_ addition [6,7].

Deposition of thick NiAl-based coatings of tens of micrometers or more, so much sought in industrial wear-resistant applications, is nowadays practically limited to the atmospheric plasma spraying (APS) deposition method. It is capable of mixing various chemical elements, but simultaneously produces coatings filled predominantly with flattened droplets crystallizing at the arrival at the substrate surface. It results in the formation of numerous voids and a number of microstructure features starting from those solidified as amorphous, through fine crystalline, up to coarse crystalline ones. A newly emerging deposition method based on mechanical embedding (ME) of powder particles into metallic substrates during their mechanical milling seems to be fully capable of competing with the APS method with respect to the thickness of the deposited coatings [8,9,10,11,12,13,14]. In spite of the high surface roughness inherently connected with the mechanism of their build up, i.e., the local impact of rotating balls used in milling vials against substrate platelets, the ME coating’s microstructure should be much more uniform than those obtained with the APS method. The preliminary investigation of coatings obtained at the relatively low milling energy of 200 rpm [15] indicated that they bear similarity to gradient structures with a layer of continuous coarse crystalline material located near the substrate and well-fused aggregates of nanocrystallites separated by numerous pores located higher up. The presence of the latter makes the ME coatings similar to these obtained with the APS method. However, the most characteristic microstructure features deciding the usefulness of ME coatings in practical applications were not described as yet.

Therefore, the presently described experiment was aimed at the deposition of thick NiAl + CrB_2_ composite coatings on model nickel and stainless steel platelets through milling at the highest rotation velocity (350 rpm) realized in a Fritsch planetary ball mill. Next, the coated platelets’ microstructure was characterized using light microscopy (LM), scanning electron microscopy (SEM), and transmission electron microscopy (TEM) methods.

## 2. Experimental Procedure

The coatings were produced using NiAl (99.9% purity/45 µm average size) intermetallic powder from Goodfellow company and CrB_2_ (99.9% purity/40 µm average size) powder from Polema JSC company (Tula, Russia). Forty grams of mixed NiAl and CrB_2_ powders (85:15 weight ratio) were loaded into a stainless steel vial each time, together with four platelets (10 mm × 10 mm × 2 mm) made of nickel (~82 HV5) or steel (~273 HV5). The above operation was performed in a glove box evacuated with a rotary vane pump down to 2 Pa and filled with argon (3 N) up to 1.6 × 10^5^ Pa. Next, steel balls of 10 mm diameter were added (the ration of ball to powder weight was approximately 10:1). The loaded containers were mounted in a high-energy Fritsch mill and rotated at 350 rpm. Every 15 min the milling was stopped for 45 min, necessary to cool down the containers (the milling times given in the text refers to actual time of vial rotation).

The coating’s characterization was started with optical microscopy observation of their sections aimed at checking their thickness and general coverage. Next, an FEI (Eindhoven, The Netherlands) Quanta Dual Beam scanning electron microscopy equipped with an EDAX energy dispersive X-ray spectroscopy (SEM/EDS) system was used to assess a distribution of CrB_2_ particles in the NiAl matrix. Finally, the microstructure investigation, including the determination of the size of crystallites forming the coatings were performed using a Tecnai G2 F20 (FEI, Eindhoven, The Netherlands, 200 kV) transmission electron microscope equipped with an integrated EDAX energy dispersive spectroscopy (TEM/EDS) detector. Thin foils from the substrate/coating interface for TEM observations were cut out using an FEI Quanta dual beam focused ion beam stand.

## 3. Results

**Figure 1:**
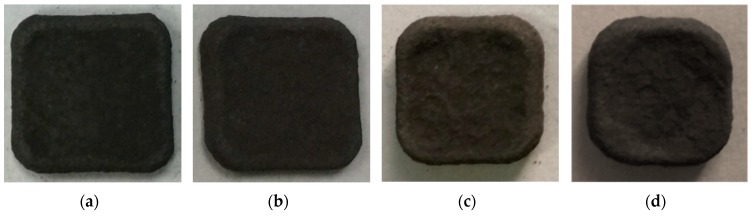
Images of nickel substrates milled with NiAl and CrB_2_ powders for: (**a**) 4, (**b**) 8, (**c**) 16, and (**d**) 32 h.

**Figure 2:**
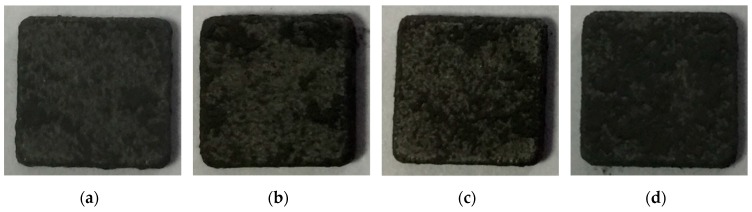
Images of steel substrates milled with NiAl and CrB_2_ powders for: (**a**) 4, (**b**) 8, (**c**) 16, and (**d**) 32 h.

The ball milling of nickel platelets with the NiAl and CrB_2_ powders at very fast speeds affects their geometry by clearly visibly rounding their edges even after 4 h of such processing (Figure 1). Extending of the milling time up to 16 and 32 h makes these changes even more pronounced. Simultaneously, the described changes in the platelets’ geometry correspond to increasing the unevenness of their surface. The approximate assessment of the coverage of the platelets’ surface with the imbedded material showed that, after 4 and 8 h of milling, large parts of the substrate were still coating-free. The nearly full coverage was first noted after 16 h, but evident coverage of all platelets’ areas was achieved only after 32 h of milling. The same processing of stainless steel platelets also caused rounding of their edges, but to a much lesser extent after corresponding time intervals (Figure 2). The roughness and coverage of the surface of steel platelets was similar to that of nickel ones but retarded in a comparable way as the rounding of their edges, i.e., their surface could be classified only as nearly fully coated after 32 h of such processing.

**Figure 3:**
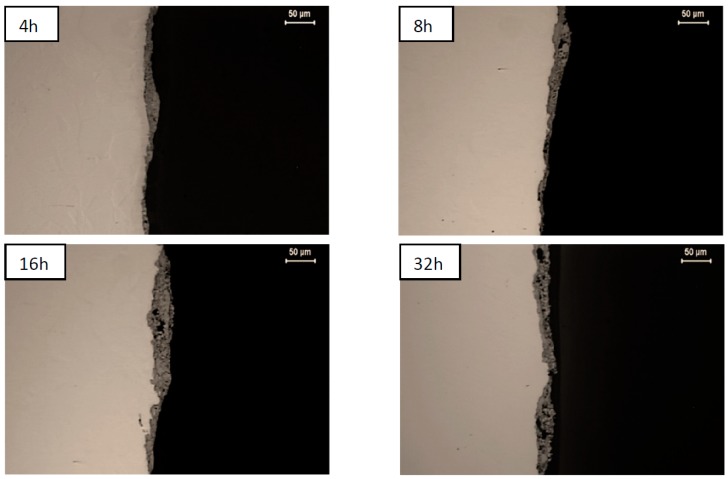
Images of composite coatings on nickel substrates milled for 4, 8, 16, and 32 h.

**Figure 4:**
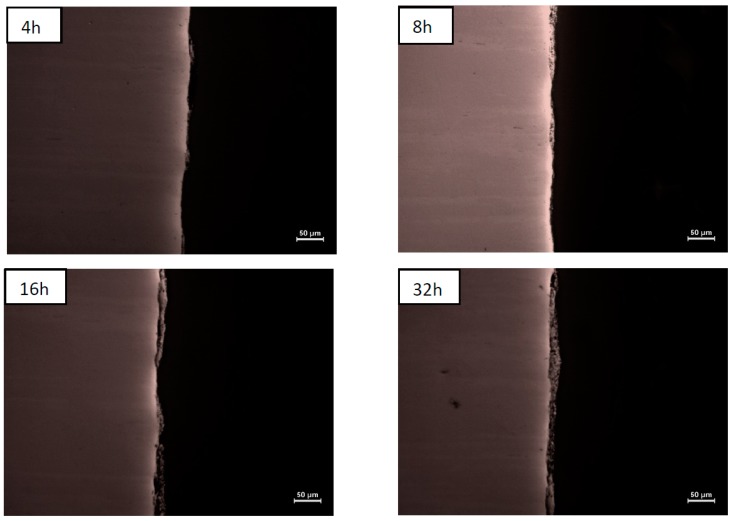
Images of composite coatings on steel substrates milled for 4, 8, 16, and 32 h.

Optical microscopy observations of sections of platelets confirmed that the coverage of their surfaces is discontinuous up to 4 and 16 h of milling, for nickel and steel material, respectively (Figure 3 and Figure 4). The longer milling times indeed allowed for their full coverage producing coatings of average thickness increasing steadily, even as their local thickness and porosity vary to a large extent (Table 1).

**Figure 5:**
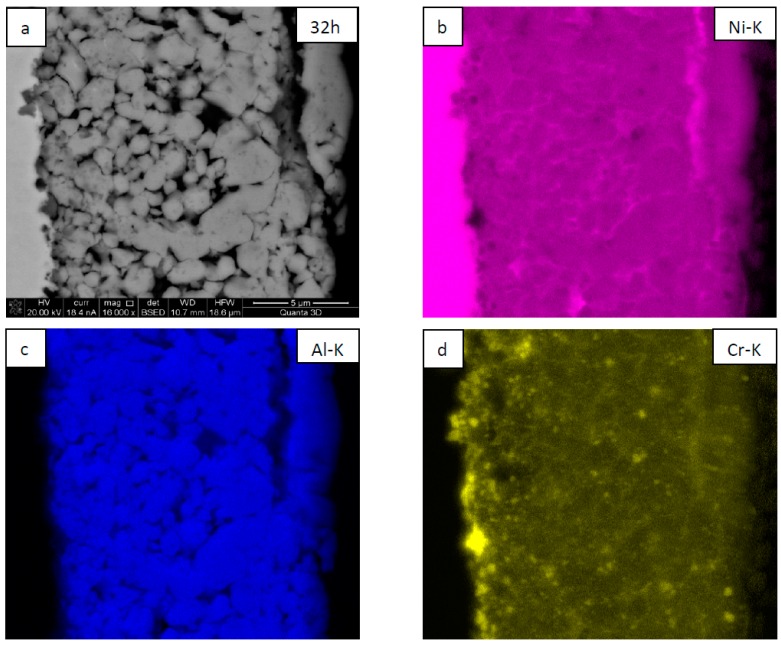
SEM/BSE image of NiAl + CrB_2_ coatings deposited on nickel substrate after 32 h of milling (**a**) and maps presenting the distribution of nickel (**b**), aluminum (**c**), and chromium (**d**).

**Figure 6:**
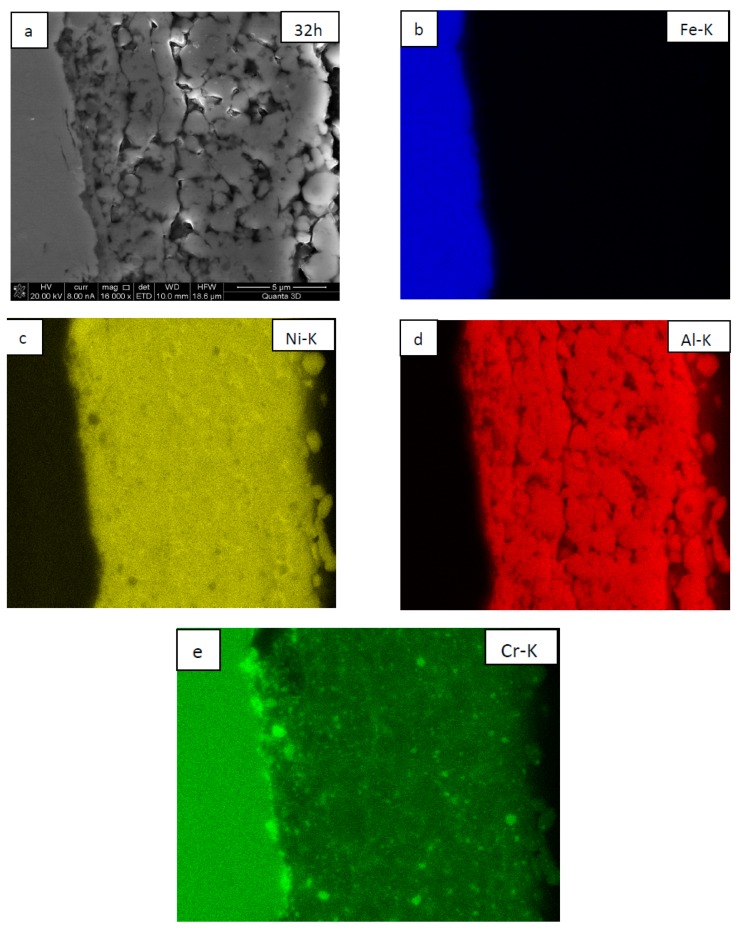
SEM/BSE image of NiAl + CrB_2_ coatings deposited on steel substrate after 32 h of milling (**a**) and maps presenting the distribution of iron (**b**), nickel (**c**), aluminum (**d**), and chromium (**e**).

SEM observations showed that the coatings are built of globular grains with numerous pores located mostly at their triple points and, to a lesser extent, at their boundaries (Figure 5a and Figure 6a). The globular grains consisted of NiAl matrix with numerous embedded fine CrB_2_ particles of slightly more grayish contrast (SEM/BSE), as confirmed separately by SEM/EDS maps presenting the local chemical distribution of elements (the position of CrB_2_ are represented in this case by the local increase of Cr intensity as the boron signal is, to a large extent, absorbed in such a material) (Figure 5b–d and Figure 6b–e). Comparing the coating deposited on nickel and steel substrates one may point out that the rounded grains of which they are built are approximately of the same size. However, more large CrB_2_ crystallites are located at the Ni/NiAl + CrB_2_ interface than at the steel/NiAl + CrB_2_ one.

**Figure 7:**
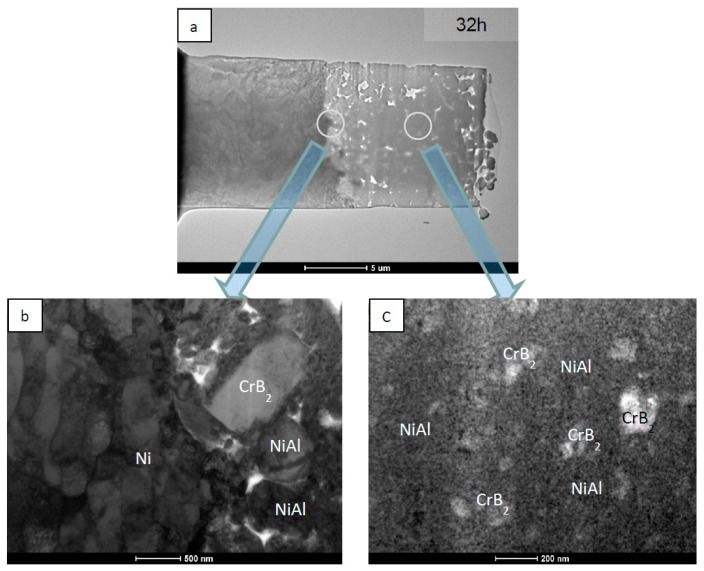
TEM images of NiAl + CrB_2_ coating deposited on nickel substrate after 32 h of milling: (**a**) general overview, (**b**) substrate/coating interface, and (**c**) center of the coating.

TEM investigations of the coatings deposited on the nickel substrates helped to establish that even after 32 h of milling some larger CrB_2_ crystallites of size approaching 0.5 µm, or even more, are either pushed into the Ni substrate or adhere to it, surrounded by NiAl particles similar in size (Figure 7a,b). The Ni substrate adjoining to the interface was highly deformed as documented by the presence of dislocation cells elongated up to ~1 µm. The interface itself is strongly corrugated with upheavals reaching up to ~0.5 mm. The globular particles filling in the interior of the coating were characterized by the presence of numerous fine CrB_2_ crystallites immersed in a nanocrystalline NiAl matrix (Figure 7a,c).

**Figure 8:**
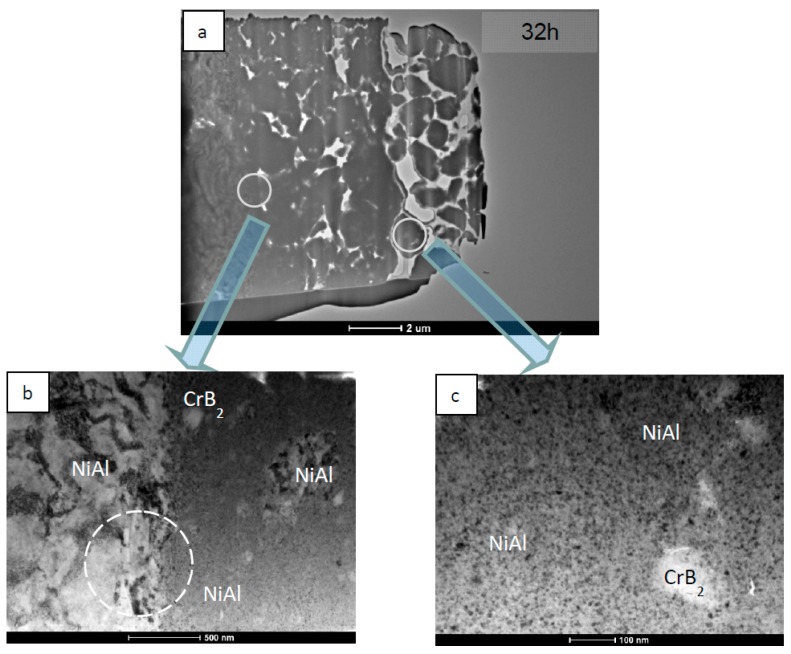
TEM images of NiAl + CrB_2_ coating deposited on steel substrate after 32 h of milling: (**a**) general overview, (**b**) coating close to the substrate, and (**c**) center of the coating.

**Figure 9:**
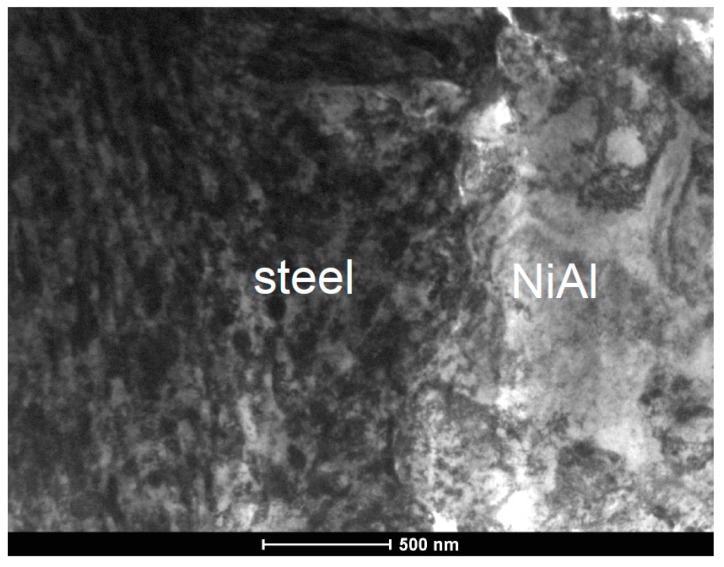
TEM image of the NiAl + CrB_2_ coating deposited on steel substrate after 32 h, presenting the substrate/coating interface.

TEM investigations of coatings deposited on stainless steel substrate at the same milling time of 32 h showed that in such a case the plastically-deformed zone extends only down to 100–200 nm from the interface (Figure 8). Additionally, the steel/NiAl + CrB_2_ interface turned out quite smooth with waviness below ¼ µm. Simultaneously, from the coating side, the interface is lined with a continuous and dense nanocrystalline layer, of average crystallite size only 2–3 times higher than that forming globular agglomerates in the rest of the coating. Occasionally, larger NiAl grains stick to steel platelets serving as a substitute substrate free from CrB_2_ particles. In such a case the impact of the rotating balls causes the formation of elongated dislocation cells at their surfaces as in Figure 8a (the area marked with broken circle). The near surface area of the steel substrate is again characterized by the presence of numerous fine grains formed under the action of steel balls (before being shielded by softer NiAl powder particles) (Figure 9).

## 4. Discussion

Mechanical synthesis (MS) of materials is, nowadays, an established method of obtaining fine crystalline or even amorphous alloys from elementary powders, which are otherwise difficult to produce due to a lack of mutual solubility [16]. Simultaneously, milling of alloy and ceramic powders turned out to be an efficient way of uniform distribution of the ceramic phase in a metallic matrix. One of the most serious problems during such processing turned out to be a sticking of the milled materials to the walls of vials used for this purpose, which could be alleviated through the addition of stearic acid or alcohol (process control agent, PCA). As in the case of some materials like Ti, Ni, or Al, accumulation of milled powders on the milling container walls was found to be quite heavy [17,18], so it could be accepted as an alternative deposition method of coatings. Papers in this area published so far describe such a possibility as the formation of coatings by mechanical alloying [8,9,10,11,12], mechanical milling [13,19] or mechanical attrition treatment [20,21]. The first one is the most popular as it points toward tools used for their production, though it did not refer to the mechanism of coating deposition and, in the case of their formation from alloyed powders, it could be misleading (none of alloying processes are involved). The coating deposition by mechanical attrition would be more appropriate if not for its close connection with attrition mills, suggesting a limitation to one special type of equipment used for powder processing. Therefore, the mechanical embedding (ME) method seems to be not only the most general but also gives readers a direct reference to the deposition mechanism.

Deposition of the coating on metallic platelets using the ME method depends first of all on the transfer of kinetic energy of rotating balls to powder particles. Therefore, it has a great deal in common with the mechanical synthesis of alloys. The efficiency of these processes depends on the same set of external parameters, including the speed of vial rotation, weight of the balls, intervals between processing runs intended for cooling the vials, and similar. The first experiments on obtaining NiAl(CrB_2_) coatings from NiAl and CrB_2_ powders were performed in a high energy mill but run at the relatively low speed of 200 rpm, helping to produce nanocrystalline thick coatings with a gradient zone close to the interface with the substrate [15]. Raising the rotation speed to 350 rpm, as in the present experiment, increased the transfer of the steel balls’ kinetic energy by four-fold [22] and resulted in an acceleration of the deposition rate through the first stage of this process, i.e., up to 8 h. However, later the buildup of deposited material was found comparable as it eventually achieves its maximum values at 20–30 µm. The above indicates that the energy transfer is effective in pressing in the still meso-, or at most the micro-crystalline, powder particles into the soft metallic substrate, which takes place only at the beginning of such processing. With time the added particles of nanocrystalline material produced during milling are also deposited forming a hard porous coating, which effectively shields the substrate. It is the brittleness of the latter which limits the coating’s final thickness.

Substrates usually need special treatments to secure acceptable coating adhesion and, in the case of APS a grit blasting of metallic plates, is a treatment of choice. It allows not only to clean, but also to roughen them, helping to form mechanical binding between the substrate and coating [23]. The balls revolving in the vial at the beginning of ME also hit non-covered substrate platelets, being a kind of grit blasting process. The locking of the substrate and coating is, however, better in MB than APS as in the latter most particles are liquid and they just splash on the roughened surface, while in the former they are pressed into the substrate as was documented for the NiAl + CrB_2_ coating on Ni substrate produced even at 200 rpm [15]. Increasing the speed of the revolving vials up to 350 rpm resulted not only in much faster rounding of the platelets’ edges but also in extending the intermixing zone of the substrate and coating material. On the other hand steel platelets milled with equally high speed showed comparable wear of edges as the nickel ones rotated at a lower speed. However, the thickness of the coatings accumulated after the longest of the applied processing times were comparable for both types of substrate. The above indicates that the type of substrate affects the size of the intermixing zone, but not the coating’s overall thickness.

## 5. Summary

Experiments with the deposition of composite NiAl(CrB_2_) coatings with the ME method using a higher speed of revolution (350 rpm against 200 rpm applied in our previous experiment) of milling containers and changing substrates from nickel to stainless steel ones proved that:

- increasing the energy transfer from steel balls to particles pressed against substrates widens the intermixing zone and raises the deposition rate of the coatings, but only at the early stage of this process;
- substituting nickel with a harder substrate, like stainless steel, narrows the coating intermixing zone, but its deposition rate remains roughly the same; and
- the coating final thickness is limited by the brittleness of the milled nanocrystalline NiAl matrix and remains approximately the same for the investigated milling parameters.

## Figures and Tables

**Table 1:** Average thickness of coatings on nickel and steel substrates obtained after various milling times.

NiAl + CrB_2_ Coatings	Milling Time (h)			
	4	8	16	32
Ni substrate	9.8 ± 3.5 µm	14.6 ± 5.5 µm	21.0 ± 10 µm	21.6 ± 6.3 µm
Steel substrate	7.8 ± 3.9 µm	8.9 ± 4.4 µm	13.5 ± 4.0 µm	19.2 ± 9.8 µm
